# Investigating the effects of platelets, platelet releasate and aspirin on colorectal cancer cell proliferation, migration and invasion

**DOI:** 10.1007/s12032-026-03264-z

**Published:** 2026-02-07

**Authors:** David Capraro, David Ewan Connor, Joanne Emily Joseph

**Affiliations:** 1 Blood, Stem Cell and Cancer Research Laboratory, St Vincent’s Centre for Applied Medical Research, St Vincent’s Hospital Sydney, Darlinghurst, Australia; 2 School of Clinical Medicine, Faculty of Medicine & Health, St Vincent’s Healthcare Clinical Campus, University of New South Wales, Sydney, Australia; 3 Sydpath, St Vincent’s Hospital Sydney, Darlinghurst, Australia

**Keywords:** Platelets, Platelet releasate, Aspirin, Colorectal cancer

## Abstract

The aims of this study were to investigate the binding of platelets to colorectal cancer (CRC) cells, platelet activation by CRC cells, and determine the effect of platelets and platelet releasate containing extracellular vesicles on CRC cell proliferation, migration and invasion following aspirin pretreatment. Platelets from healthy individuals were pretreated with aspirin before co-incubation with CRC cells. Platelet activation by CRC cells was analysed by flow cytometry and the binding of platelets to CRC cells investigated using immunofluorescence microscopy. Platelet releasate containing extracellular vesicles was generated by centrifugation. The effect of platelets and platelet releasate on CRC cell proliferation was investigated using carboxyfluorescein succinimidyl esterand dimethylthiazol-carboxymethoxyphenyl-sulfophenyl-tetrazolium assays. Migration and invasion were examined using Transwell assays. HCT15 and HCT116 CRC cells activated platelets, with higher numbers of CRC cells activating more platelets, however aspirin was unable to inhibit platelet activation. Platelets are also able to bind to HCT15 and HCT116 cells. Platelets and platelet releasate did not affect HCT15 and HCT116 proliferation, however, aspirin directly inhibited the proliferation of both cell lines. Platelets and platelet releasate significantly increased the migration and invasion of HCT116, but not HCT15 cell lines. Platelets adhere to and are activated by CRC cells lines, with aspirin unable to inhibit this activation. CRC proliferation was not affected by platelets, however the effect on cancer cell migration and invasion was dependent upon the cell line, suggesting the effects of platelets and platelet releasate containing extracellular vesicles may be patient specific. The inhibitory effect of aspirin on colorectal cancer proliferation is a direct effect on the cancer cell and independent of platelets and extracellular vesicles.

**Supplementary Information:**

The online version contains supplementary material available at 10.1007/s12032-026-03264-z.

## Introduction

Colorectal cancer (CRC), also known as bowel cancer, is overall the fourth most common cancer and the second highest cause of cancer death in Australia [1]. Early retrospective analyses of large cohort studies and randomised control trials have found that long-term regular aspirin use is associated with significantly reduced CRC incidence, mortality and metastasis [2–5]. Since this time, subsequent prospective randomised clinical trials have produced conflicting data, with CRC rates and long-term survival of patients being poorer for those receiving aspirin [6, 7]. The mechanisms that contribute to the chemo-preventative effect of aspirin on CRC are currently unclear and may potentially be derived from its antiplatelet effect [8].

It is well established that platelets, extracellular vesicles and cancer have a complex and interconnected relationship. Platelets were first directly associated with cancer in 1872, with malignant tumours associated with elevated platelet counts [9]. This negative prognostic indicator has since been reported in various advanced and metastatic cancers [10]. In 1968, reduced platelet counts in mice were associated with reduced metastasis of mammary tumour cells [11]. Platelet aggregation is induced in vitro by tumour cells [10], with tumour-induced platelet aggregation correlated to in vivo tumour metastasis [12, 13].

Proteomic analysis of platelet releasate has detected over 1,300 proteins including transforming growth factor β (TGF-β) and platelet-derived growth factor D (PDGF-D) which can both promote epithelial-to-mesenchymal transition (EMT) [14–16]. Platelet releasate can also enhance the proliferation of gastric cancer cell lines, with this enhancement inhibited by the addition of aspirin [17]. Stomach tumours in mice demonstrate slower tumour growth and significantly decreased tumour size when treated with oral aspirin or an antiplatelet antibody [17]. HT29 CRC cell lines co-incubated with platelets undergo EMT and the pretreatment of platelets with aspirin inhibited EMT [18]. When injected into mice, co-incubated HT29 cells cause a higher incidence of lung metastasis compared to control HT29 cells, with this increased metastasis reduced when mice were given daily oral aspirin [18].

The aims of this study were to investigate the in vitro binding of platelets to CRC cells, stimulation of platelet activation by CRC cells, and the effect of platelets and platelet releasate on CRC cell proliferation, migration and invasion following aspirin treatment.

## Materials and methods

### Ethical approval

This study was performed in line with the principles of the Declaration of Helsinki. Ethical approval was obtained from the St Vincent’s Hospital Human Research Ethics Committee (HREC/17/SVH/76 and 2023/ETH00384). Informed consent was obtained from all individual participants included in the study.

### Materials

HCT15 and HCT116 human colorectal cancer cells (ATCC, Manassas, USA), RPMI 1640 + L-glutamine, fetal bovine serum, penicillin/streptomycin and Dulbecco’s phosphate buffer saline (Gibco, Waltham, USA); Vacuette tube 9mL ACD-B (Greiner Bio-One, Kremsmünster, Austria); prostaglandin I_2_ (PGI_2_) sodium salt (Cayman Chemicals, Ann Arbor, USA); thrombin receptor activating peptide (Roche, Basel Switzerland); CD41 antibody (Abcam, Cambridge, UK); EpCAM (D4K8R) XP antibody, anti-mouse IgG (H + L), F(ab’)2 fragment Alexa Fluor 488, anti-rabbit IgG (H + L), F(ab’)_2_ fragment Alexa Fluor 555 and ProLong Gold Antifade Reagent with DAPI (Cell Signalling Technology, Danvers, USA); Vacutainer Citrate 2.7mL tubes, TruCount tubes, CD42b PE, CD62p APC, CD63 PE-Cy7 and PAC-1 FITC antibodies (BD Biosciences, Franklin Lakes, USA); paraformaldehyde 16% solution, EM grade (Electron Microscopy Sciences, Hatfield, USA); sodium chloride 0.9% for irrigation (Baxter, Deerfield, USA); CellTrace Carboxyfluorescein succinimidyl ester (CFSE) Cell Proliferation Kit (Thermo Fisher Scientific, Waltham, USA); cell culture plates, Transwell 6.5 mm 8.0 μm polycarbonate insert, Matrigel and 5mL polystyrene round-bottom Tube 12 × 75 mm (Corning, Corning, USA), crystal violet solution, aspirin and phenazine methosulfate (PMS) (Sigma-Aldrich, St. Louis, USA). CellTiter 96(R) AQueous 3-(4,5-dimethylthiazol-2-yl) −5- (3-carboxymethoxyphenyl) −2- (4-sulfophenyl)−2 H-tetrazolium (MTS) Reagent Powder (Promega, Madison, USA).

### Cell culture

HCT15 and HCT116 human CRC cell lines were cultured in RPMI Medium 1640 with L-glutamine supplemented with 10% fetal bovine serum (FBS), 100U/mL penicillin and 100µL/mL streptomycin at 37 °C and 5% CO_2_.

### Platelet washing

The platelet washing method used was adapted from Ambrose et al. [19]. Whole blood collected in two ACD-B vacutainer tubes from healthy individuals with no history of cancer was centrifuged at 160*g* for 20 min with a brake. The platelet-rich plasma (PRP) was transferred to a new tube, PGI_2_ was added (final concentration 200ng/mL) to prevent artefactual platelet activation and centrifuged at 600*g* for 15 min with a brake. The platelet pellet was resuspended in Dulbecco’s PBS (DPBS), PGI_2_ added and centrifuged again at 600x*g* for 15 min. The final platelet pellet was resuspended with DPBS. The number of platelets isolated was counted by flow cytometry using Trucount tubes. The platelets were then pretreated with DPBS or aspirin at a final concentration of 1mM for 30 min.

### Generation of platelet releasate

To generate platelet releasate, platelet aliquots were activated using thrombin receptor activating peptide (TRAP) at a final concentration of 80µM for 30 min at room temperature. The platelets were pelleted by centrifugation at 2,500*g* for 15 min with a brake, the releasate carefully transferred to a new tube before repeating the centrifugation and transferring the releasate to a second new tube.

### Immunofluorescence microscopy

To examine platelet binding to HCT15 and HCT116 CRC cells, 1 × 10^6^ platelets were co-incubated with 3 × 10^5^ CRC cells for 24 h (*n* = 5) in a cell culture plate. The platelets and CRC cells were stained with CD41 and EpCAM primary antibodies and IgG Fab Alexa AF488 and AF555 secondary antibodies. ProLong Gold Antifade Mountant with DAPI was used to stain the nuclei of the CRC cells. Cells were imaged using a Leica DM5500 immunofluorescence microscope.

### Platelet activation flow cytometry

The ability of CRC cells to activate platelets and aspirin to inhibit platelet activation was investigated using flow cytometry. Whole blood collected in sodium citrate vacutainer tubes from healthy individuals (*n* = 5) was incubated with 1mM aspirin or DPBS as a control for 30 min. In 5mL polystyrene round-bottom tubes, 5µL of treated whole blood was incubated with 5µL CD42b PE, 5µL CD62p APC, 2.5µL CD63 PE-Cy7, 2.5µL PAC-1 FITC and a 25µL cell suspension containing 1,000, 10,000 or 100,000 HCT15 or HCT116 cells. Separate control tubes containing fluorophore conjugated isotype controls were also prepared. After 30 min of incubation at room temperature, the samples were fixed using 1mL of 0.2% paraformaldehyde in 0.9% sodium chloride. Flow cytometry was performed using a BD LSRFortessa X-20 flow cytometer, with platelets identified using forward and side scatter gating on CD42b + events. The gating strategy is shown in Supplementary Fig. 1.

### Transwell migration and invasion assay

The effect of platelets and platelet releasate on CRC cell migration and invasion was examined using Transwell inserts with 8 μm pores placed into 24-well cell culture plates. For invasion assays, Transwell inserts were coated with 500 µg/mL Matrigel. For each well, 1 × 10^5^ HCT15 or HCT116 cells in serum-free cell culture media and 5 × 10^7^ platelets or the releasate from 5 × 10^7^ platelets were added to the upper Transwell insert (*n* = 5). Complete cell culture media with 10% FBS as a chemoattractant was added to the lower chamber of each well. Following incubation at 37 °C and 5% CO_2_ for 24 h, CRC cells passing though the insert membrane were fixed with 4% paraformaldehyde and stained with crystal violet before being counted with a light microscope. The number of cells counted for each treatment was compared to the control.

### CFSE proliferation assay

The effect of platelets and platelet releasate on CRC cell proliferation was investigated using a CFSE proliferation assay. HCT15 and HCT116 cells were stained with CFSE at a final concentration of 5µM. 2.5 × 10^4^ CFSE stained cells were then seeded into 12-well cell culture plates. After 24 h, the CRC cells were incubated with 2.5 × 10^7^ platelets or the releasate from 2.5 × 10^7^ platelets and co-incubated for 48 h at 37 °C and 5% CO_2_ (*n* = 6). After 48 h, the CRC cells were liberated and analysed using flow cytometry. The proliferation index was calculated using the cell tracking function of ModFitLT (version 5.0.9). The proliferation index was divided by the proliferation index of the control to determine the fold change.

### MTS proliferation assay

2 × 10^3^ HCT15 and HCT116 CRC cells were seeded into 96-well cell culture plates. After 24 h, each well was treated with different numbers of platelets, aspirin treated platelets (*n* = 5) or 1mM aspirin (*n* = 10). After 48 h of co-incubation at 37 °C and 5% CO_2_, 333 µg/mL MTS and 25µM PMS solution was added to the wells and incubated at 37 °C for 2 h in the dark. The absorbance at 490 nm was measured using a FLUOstar Omega microplate reader (BMG Labtech). The background absorbance (620 nm) was subtracted from each well. A corrected absorbance was calculated by subtracting the absorbance of the control wells.

### Statistical analysis

Statistical analysis was performed using Prism software (v9.0.1, GraphPad, San Diego, USA). Unless otherwise stated, data is presented as the mean with error bars representing the standard error of the mean. Paired data was analysed using a paired t-test. Unpaired data was analysed with a Mann-Whitney test. Data is presented as box whisker plots showing the 5th and 95th percentiles.

## Results

### The effect of aspirin on platelet activation by CRC cells

**Fig. 1:**
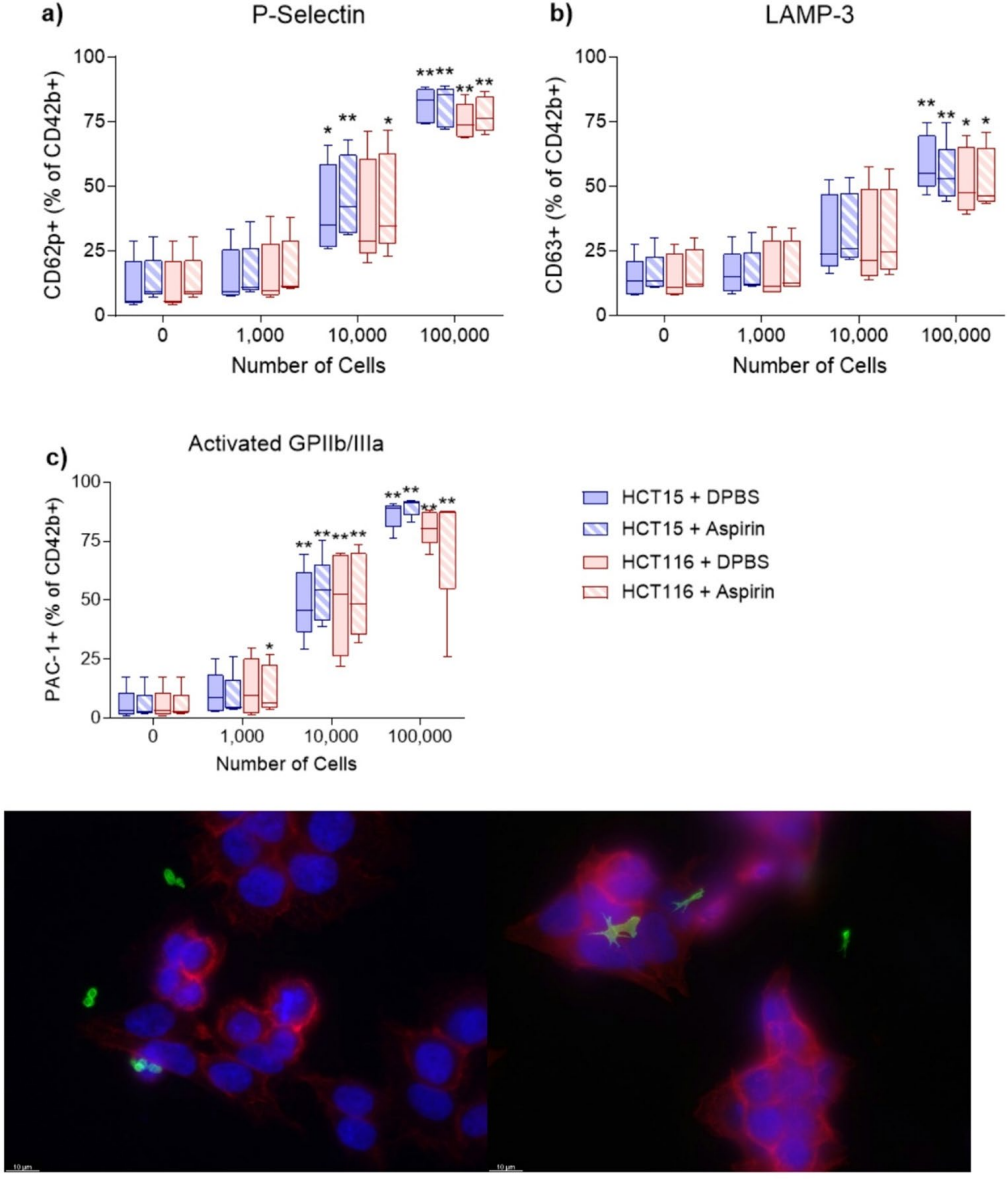
Platelet activation and binding to colorectal cancer cells. Whole blood obtained from healthy individuals (*n* = 5) was treated with DPBS or 1mM aspirin before incubation with different concentrations of HCT15 or HCT116 colorectal cancer cells. Platelet activation was measured using flow cytometry for the platelet activation markers (**a**) P-selectin, (**b**) LAMP-3 and (**c**) activated GPIIb/IIIa. Representative images of platelets binding to d) HCT15 and e) HCT116 cells. Platelets were stained using an anti-CD41 antibody (green) and CRC cells were stained using an anti-EpCAM antibody (red) and DAPI (blue) and imaged using immunofluorescence microscopy. Data presented as box whisker plots for *n* = 5 experiments. * *P* < 0.05, ** *P* < 0.01

Platelets incubated in the presence of increasing numbers of CRC cells demonstrated a concentration dependent increase in platelet activation for all three activation markers (P-selectin, LAMP-3 and activated GPIIb/IIIa). There was no significant difference between HCT15 and HCT116 stimulated platelet activation and the pretreatment of whole blood with 1mM aspirin had no effect on platelet activation (Fig. 1). On immunofluorescence microscopy, platelets were able to bind to HCT15 and HCT116 cells (Fig. 1).

### The effect of platelets and platelet releasate on CRC cell migration and invasion

**Fig. 2:**
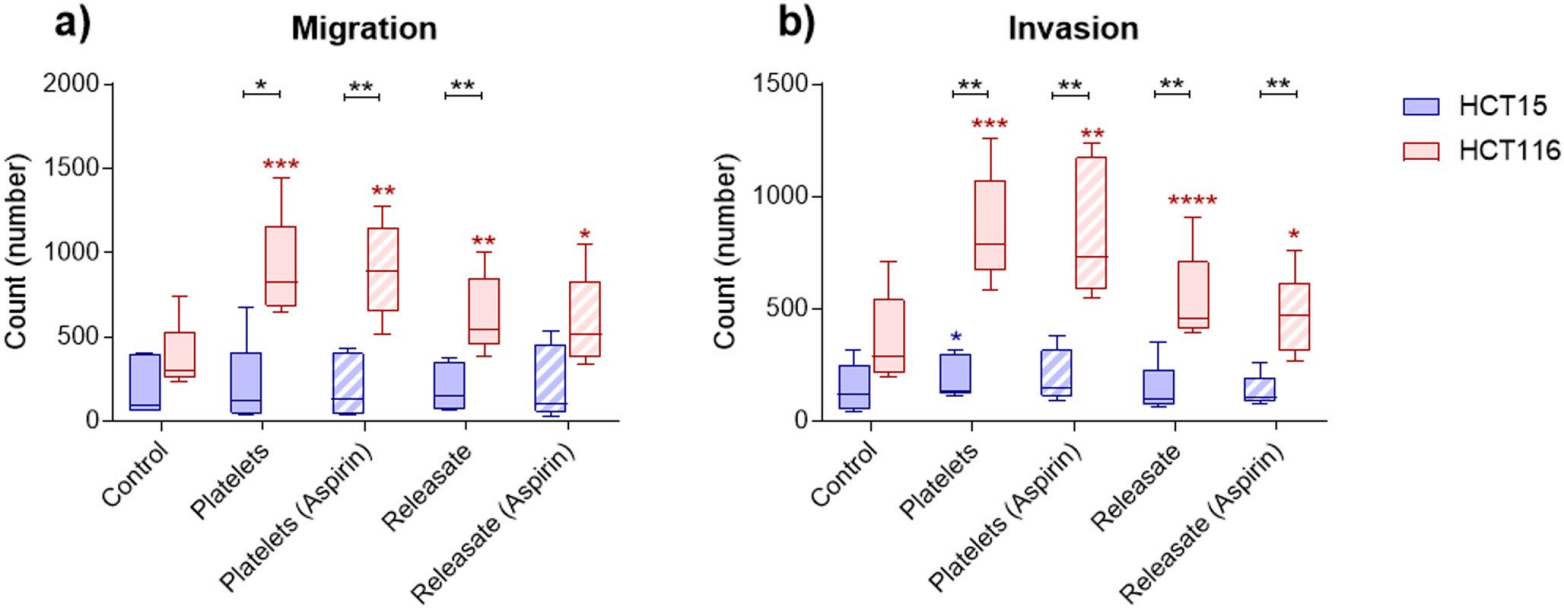
The effect of platelets and platelet releasate on colorectal cancer cell migration and invasion. Inserts in transwell assays were (**a**) uncoated to measure migration or (**b**) Matrigel coated to measure invasion of HCT15 and HCT116 cells co-incubated with platelets or platelet-releasate from healthy individuals (*n* = 5) for 24 h. Platelets were pretreated with DPBS or 1mM aspirin and platelet releasate was generated from platelets pretreated with DPBS or aspirin. Data is presented box whisker plots for *n* = 5 experiments. * *P* < 0.05, ** *P* < 0.01, *** *P* < 0.001, **** *P* < 0.0001

Co-incubation of HCT116 cells with platelets or platelet releasate significantly increased both the migration and invasion of cells when compared to control (Fig. 2). Co-incubation of HCT15 cells with platelets or releasate did not significantly increase migration and only resulted in a limited but significant increase in invasion for HCT15 cells co-incubated with platelets. There was no significant effect of aspirin on inhibiting migration or invasion of cells co-incubated with platelets or platelet releasate. There was a significant difference between migration and invasion of HCT116 and HCT15 cells co-incubated with platelets and platelet releasate.

### The effect of platelets, platelet releasate and aspirin on CRC cell proliferation

**Fig. 3:**
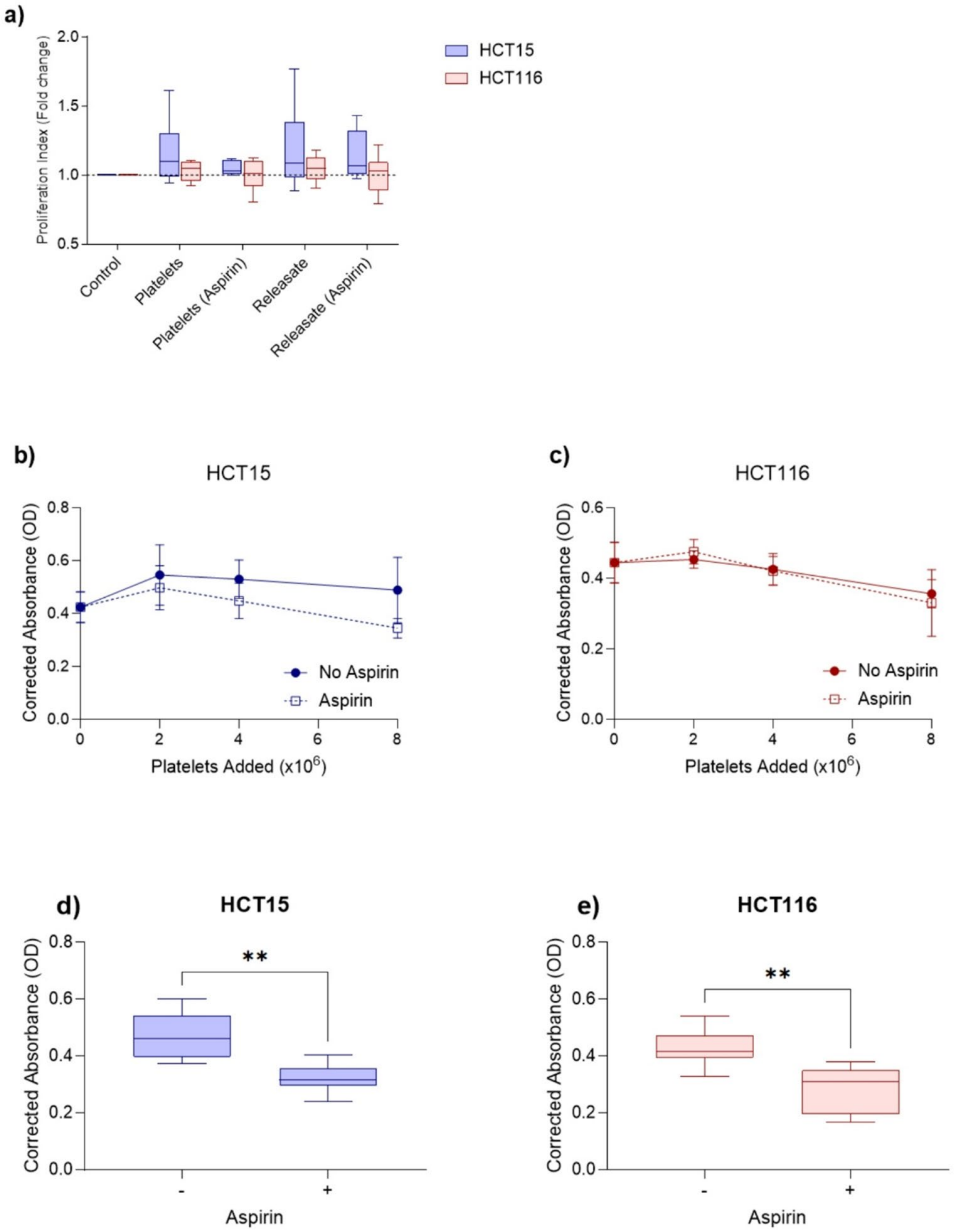
The effect of platelets and platelet releasate on colorectal cancer cell proliferation. **a**) HCT15 and **b**) HCT116 cells stained with CFSE were co-incubated with platelets or platelet releasate from healthy individuals (*n* = 6) for 48 h. Platelets were pretreated with DPBS or 1mM aspirin and platelet releasate was generated from platelets pretreated with DPBS or aspirin. The cells were analysed using flow cytometry and ModFit LT software which calculated a proliferation index. Data is presented box whisker plots for *n* = 6 experiments. (**c**) HCT15 and (**d**) HCT116 cells were co-incubated for 48 h with platelets from healthy individuals (*n* = 5) pretreated with DPBS or 1mM aspirin, with proliferation determined using MTS. Data is presented as the mean with error bars representing the standard error of the mean for *n* = 5 experiments. (**e**) HCT15 and (**g**) HCT116 cells were incubated with 1mM aspirin for 48 h (*n* = 10) and the effect it had on proliferation was determined using MTS proliferation assays. Data is presented box whisker plots for *n* = 10 experiments. ** *P* < 0.01

Platelets and platelet releasate did not have a significant effect on HCT15 and HCT116 proliferation using either the CFSE or MTS assay (Fig. 3). Treating the platelets with 1mM aspirin did not influence the effect of platelets or platelet releasate on CRC cell proliferation. While aspirin-treated platelets did not affect CRC proliferation, the direct effect of aspirin addition to HCT15 and HCT116 cell cultures demonstrated significant inhibition of the proliferation of both cell lines.

## Discussion

Long-term regular aspirin has been associated with significantly reduced CRC incidence, mortality and metastasis [2–5], however more recent trials have produced conflicting data, with colorectal cancer rates and long-term survival of patients being poorer for those receiving aspirin [6, 7]. The mechanisms that contribute to this effect are currently unclear but may derive from its antiplatelet effect [8].

In this study we found platelets were able to bind to HCT15 and HCT116 CRC cells, with HCT15 and HCT116 cells stimulating the activation of platelets in a concentration dependent manner. Platelets and platelet releasate did not affect HCT15 and HCT116 proliferation. Co-incubation of platelets and platelet releasate significantly increased the migration and invasion of HCT116, but not HCT15 cells. Aspirin pretreatment of platelets had no effect on platelet activation, platelet binding or on HCT15 and HCT116 proliferation, migration or invasion, however, aspirin was able to directly inhibit HCT15 and HCT116 proliferation.

Inherent differences between the two cell lines could potentially explain the discrepancy between the migration of HCT116 and HCT15. HCT15 cells were sourced from a patient with Dukes’ C (similar to TNM stage III) CRC [20], while the staging of HCT116 has been reported in separate publications as either Dukes’ A (stage I) [20] and Dukes’ D (stage IV) [21]. While both cell lines possess microsatellite instability and mutations in *KRAS* and *PIK3CA*, HCT15 possesses a *TP53* mutation [21] and HCT116 potentially possesses a *BRAF* mutation [21, 22]. Mechanical and structural differences between the two cell lines are a likely reason why HCT116 cells were more migratory than HCT15 cells. Brás et al. correlated the biomechanical properties, cell structure and migratory behaviour of three CRC cell lines including HCT15 and HCT116. They found that HCT116 cells were more migratory than HCT15 cells and had more cortical F-actin, vinculin, focal adhesions and filopodia formation but less tubulin [23].

The promotion of cancer cell migration and invasion by platelet releasate and platelet-derived extracellular vesicles (PEVs) has previously been demonstrated in various cell lines. While PEVs are not a direct comparison to platelet releasate, they are present in platelet releasate as one of the potential contributors to the effect platelets have on cancer cell migration and invasion. Co-incubation with PEVs significantly increased the migration of MDA-MB-231 breast cancer cells and the addition of 200µM aspirin significantly inhibited this effect. However, platelets had no effect on MDA-MB-231 cells while PEVs had no effect on SKBR-3 and BT474 breast cancer cells [24]. PEVs are also able to stimulate the invasion of MDA-MB-231, BT-549 and T47D breast cancer cells [24, 25] and A549 lung cancer cells [26]. The releasate of activated platelets significantly increased MDA-MB-231 invasion compared to resting platelet releasate. Treating the platelets with 100µM aspirin before activation did not inhibit the effect of activated platelet releasate on MDA-MB-231 invasion [27].

We found that HCT15 and HCT116 CRC cells activate whole blood platelets in a concentration dependent manner with increased numbers of CRC cells causing higher platelet activation (Fig. 1). The effect of different concentrations of CRC cells on platelet activation has not previously been explored but other studies have confirmed that interactions between cancer cells and platelets result in platelet activation. HT29 CRC cells can activate platelets, alter platelet morphology and stimulate the release of PEVs [28]. MCF-7, MDA-MB-231, BT-20 and SKBR-3 breast cancer cell lines are also able to activate platelets [27]. Cancer cells activate platelets through the release of molecules such as ADP or by direct cell-cell interactions [29, 30].

Incubating whole blood with aspirin had no effect on platelet activation by HCT15 and HCT116 cells (Fig. 1) and this result has been previously reported using the same platelet activation markers. Chronos et al. examined platelet activation by flow cytometry of P-selectin, LAMP-3 and platelet-bound fibrinogen. They also found that these activation markers were unaffected in healthy individuals receiving aspirin therapy [31].

Platelets and platelet releasate did not have a significant effect on HCT15 and HCT116 proliferation (Fig. 2). Previous studies have demonstrated that platelets and platelet releasate are able to stimulate cancer cell proliferation. SW480 and SW620 CRC cells and PANC-1 pancreatic cancer cells co-cultured with resting or activated platelets had significantly increased proliferation. Pretreating platelets with 20µM aspirin significantly reduced the ability of activated platelets to promote SW480 and PANC-1 proliferation. Platelet releasate significantly increased PANC-1 proliferation while aspirin pretreatment significantly inhibited this effect [32]. Platelet releasate has also been able to significantly increase the proliferation of MCF-7 and MDA-MB-231 breast cancer cells [33] and MKN-45, NUGC-3 and AGS gastric cancer cells. The addition of 1mM aspirin significantly inhibited its effect on the MKN-45 and AGS cells [17]. Our results conflict with what has previously been published and this could potentially be caused by different cell lines having different interactions with platelets and platelet releasate. Experimental design could also have affected our results as in the CFSE assay the ratio of platelets to cancer cells used was lower what Mitrugno et al. used [32]. For the MTS assay, we included higher ratios but the co-incubation was performed in a 96-well plate and there might not have been enough space and cell culture media to support the total number of CRC cells and platelets.

While platelets and platelet releasate did not affect CRC cell proliferation, aspirin was able to directly inhibit HCT15 and HCT116 proliferation. Aspirin irreversibly inactivates cyclooxygenase (COX) and COX-2 is often overexpressed in CRC [34, 35]. However, both HCT15 and HCT116 cells do not express COX-2 [36, 37]. Our results are in concordance with what has previously been reported [38]. Zumwalt et al. reported that aspirin inhibited the growth of eight CRC cell lines including HCT15 and HCT116. This effect was greatest in cell lines that possessed *PIK3CA* mutations with the presence of aspirin also causing significant cell cycle arrest. HCT15, HCT116 and other cell lines with *PIK3CA* mutations grew faster than those without leading to Zumwalt et al. to hypothesise that rapid cell proliferation increases their sensitivity to aspirin, similar to how faster growing tumours are more sensitive to chemotherapy. The *PIK3CA* mutations could also be making the cells more sensitive to aspirin [38]. These results suggest that aspirin directly inhibits cancer proliferation and future studies can explore if this effect still occurs in the presence of platelets and platelet releasate.

There is a large amount of evidence that suggests regular use of low dose aspirin can reduce cancer incidence, metastasis and mortality, with the greatest effect observed in CRC [2–5]. Currently, the mechanisms that contribute to the anticancer effect of aspirin are unclear but it has been hypothesised that it is derived from the antiplatelet effect of aspirin [8]. This study attempted to examine that hypothesis, however, we found that aspirin can act directly on CRC cells and its anticancer effect is potentially independent from its antiplatelet effect. As a result of this it would be beneficial for future studies to include aspirin in the co-culture experiments rather than pretreating the platelets with aspirin before co-culture.

A major limitation of this study is the small sample size. Future studies need more participants and larger sample sizes. A potential limitation of this study is the pretreatment of platelets with 1mM aspirin for 30 min is not directly comparable to long-term regular aspirin use. Future studies can involve participants who regularly take low dose aspirin and compare them with control participants who do not take aspirin. Another limitation was the use of platelets from healthy individuals rather than people with CRC. The effect that platelets and platelet releasate from people with CRC have on CRC cell proliferation, migration and invasion could be different to those of healthy individuals. The direct effect of aspirin on CRC cells was also not explored in detail. Aspirin can be included in the co-cultures at varying concentrations rather than just pretreating the platelets with aspirin. Other antiplatelet agents can also be examined to determine what effect they have on CRC cells.

In summary, we found that platelets bind to HCT15 and HCT116 CRC cells and that aspirin was unable to prevent this binding. HCT15 and HCT116 cells stimulate platelet activation in a concentration dependent manner and aspirin was unable to inhibit this activation. Platelets or platelet releasate containing extracellular vesicles did not have a significant effect on HCT15 and HCT116 proliferation, but did affect migration and invasion in HCT116 cells, suggesting the effects of platelets and platelet releasate containing extracellular vesicles may be patient specific. Aspirin pretreatment of platelets had no effect on activation, proliferation, migration or invasion, but did directly inhibit the proliferation of CRC lines. The inhibitory effect of aspirin on CRC appears to be a direct effect on the cancer cell and independent of platelets and extracellular vesicles.

## Supplementary Information

Below is the link to the electronic supplementary material.

Supplementary material 1 (DOCX 575.0 kb)

## Data Availability

No datasets were generated or analysed during the current study.
