# Supplementary material for: Investigating the effects of platelets, platelet releasate and aspirin on colorectal cancer cell proliferation, migration and invasion
Source: Med Oncol. 2026 Feb 7;43(3):144. doi: 10.1007/s12032-026-03264-z (PMC12882856; doi:10.1007/s12032-026-03264-z)
Supplement: Supplementary file 1 — Supplementary material 1 (DOCX 575.0 kb) [file 12032_2026_3264_MOESM1_ESM.docx]

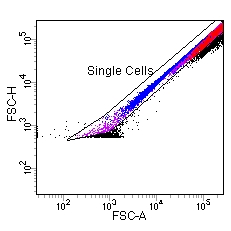


**a)**


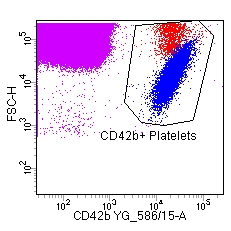


**b)**


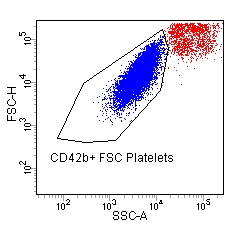


**c)**


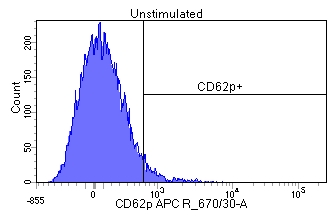


**d**)


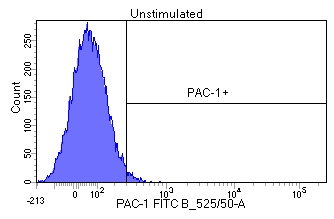


**e**)


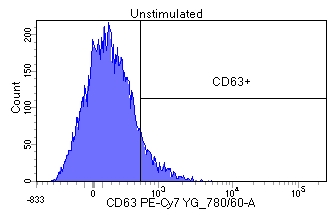


**f**)

Supplementary Figure 1. Flow cytometry gating strategy to examine platelet activation by colorectal cancer cells. The activation of whole blood platelets was determined using gating to select for (a) single cells, (b) all CD42b-positive platelet events and (c) a third gate that separated the platelets from platelet-cellular aggregates and coincident events based on size. From this gate 10,000 platelet events were recorded and the percentage of activated platelets was based on (d) APC fluorescence for CD62p, (e) FITC for PAC-1 and (f) PE-Cy7 for CD63.
